# Current Knowledge and Perspectives of Pyrrolizidine Alkaloids in Pharmacological Applications: A Mini-Review

**DOI:** 10.3390/molecules26071970

**Published:** 2021-03-31

**Authors:** Xianqin Wei, Weibin Ruan, Klaas Vrieling

**Affiliations:** 1 Institute of Entomology, College of Life Sciences, Nankai University, Tianjin 300071, China; ruanweibin@nankai.edu.cn; 2 Plant Science and Natural Products, Institute of Biology, Leiden University, Sylviusweg 72, P.O. Box 9505, 2300 RA Leiden, The Netherlands; k.vrieling@biology.leidenuniv.nl

**Keywords:** plant secondary metabolites, pharmacy, alkaloids, herb medicine, defense, regulations

## Abstract

Pyrrolizidine alkaloids (PAs) are a widespread group of secondary metabolites in plants. PAs are notorious for their acute hepatotoxicity, genotoxicity and neurological damage to humans and animals. In recent decades, the application of PAs for beneficial biological activities to cure disease has drawn greater attention. Here, we review the current knowledge regarding the pharmacological properties of PAs and discuss PAs as promising prototypes for the development of new drugs.

## 1. Introduction

Pyrrolizidine alkaloids (PAs) are an important group of secondary metabolites in plants [1,2]. More than 500 different PAs were identified in more than 6000 plant species to date [1,3]. PAs have been identified in Apocynaceae (*Alafia*, *Aristeguietia*, *Brachyglottis*, *Cacalia*, *Eupatorium*, *Ligularia*, *Pittocaulon*, *Senecio* and *Tephroseris*), Boraginaceae (*Anchusa*, *Borago*, *Cynoglossum*, *Echium*, *Heliotropium*, *Lithospermum*, *Onosma*, *Rindera* and *Solenanthus*), Convolvulaceae (*Argyreia*), Fabaceae (*Castanospermum* and *Crotalaria*), Hyacinthaceae (*Muscari* and *Scilla*), Orchidaceae (*Cremastra* and *Liparis*), and Santalaceae (*Amphorogyne* and *Osyris*) [4,5,6].

**Figure 1:**
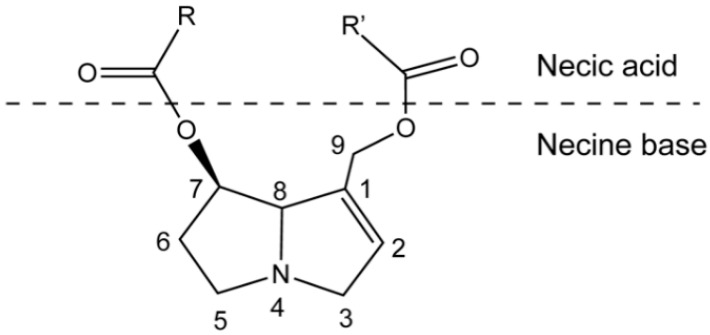
Schematic structure of pyrrolizidine alkaloid.

**Figure 2:**
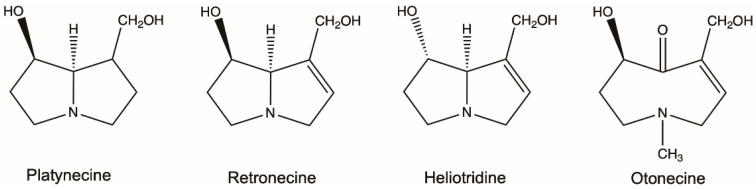
Most common necine bases in pyrrolizidine alkaloids (PAs). PAs are generally classified into four types based on the representative necine bases (platynecine, retronecine, heliotridine and otonecine).

PAs are ester alkaloids composed of a necine base (two fused five-membered rings joined by a single nitrogen atom) and a necic acid (one or two carboxylic ester arms) (Figure 1) [7]. The combination of a set of necine bases and necic acids results in a considerable amount of structural diversity in PAs. This is further amplified by modifications, including *N*-oxidation of the tertiary nitrogen of the necine base, hydroxylation of the necine base and/or the necic acid, dehydrogenation, halogenation and acetylation of hydroxy groups of the acid moiety. It is not surprising that several hundreds of different PAs have already been identified, and each year new PAs are detected. Based on the structure of the necine base, the majority of the PAs can be sorted into four groups: retronecine-type, heliotridine-type, otonecine-type and platynecine-type (Figure 2) [8,9]. Except for otonecine, the other three bases can form *N*-oxides [9]. The platynecine type of PAs contain a saturated necine base unit and are less toxic, while the other three bases contain a 1,2-unsaturated necine base unit and are highly toxic.

PAs are notorious for their serious hepatotoxicity, genotoxicity and neurological damage, affecting livestock, wildlife and humans [7,10,11]. PAs were detected in honey, milk and other food supplements [12,13,14,15,16]. However, for plants PAs play an important role in defense against herbivores and pathogens [17,18,19]. Several PA-containing plants are used in herb medicines in many countries [20,21,22,23,24,25]. For example, in India *Heliotropium subulatum* plants were used for boils, insect stings, cores, throat infections, ulcers and snake bite [26]. Anti-microbial, anti-inflammatory, anti-cancer and anti-viral effects are known from several PAs isolated from plants. In this review, we will focus on the pharmacological properties of PAs by summarizing the related papers published in the past 50 years.

## 2. Pharmacological Effects

### 2.1. Anti-Microbial Activity

Killing or inhibiting the growth of microorganisms and especially pathogenic microorganisms is defined as anti-microbial activity, and it includes anti-bacterial and anti-fungal activities [27]. PAs with anti-microbial properties were mainly isolated from the genus of *Heliotropium*, *Crotalaria* and *Senecio* plants.

**Figure 3:**
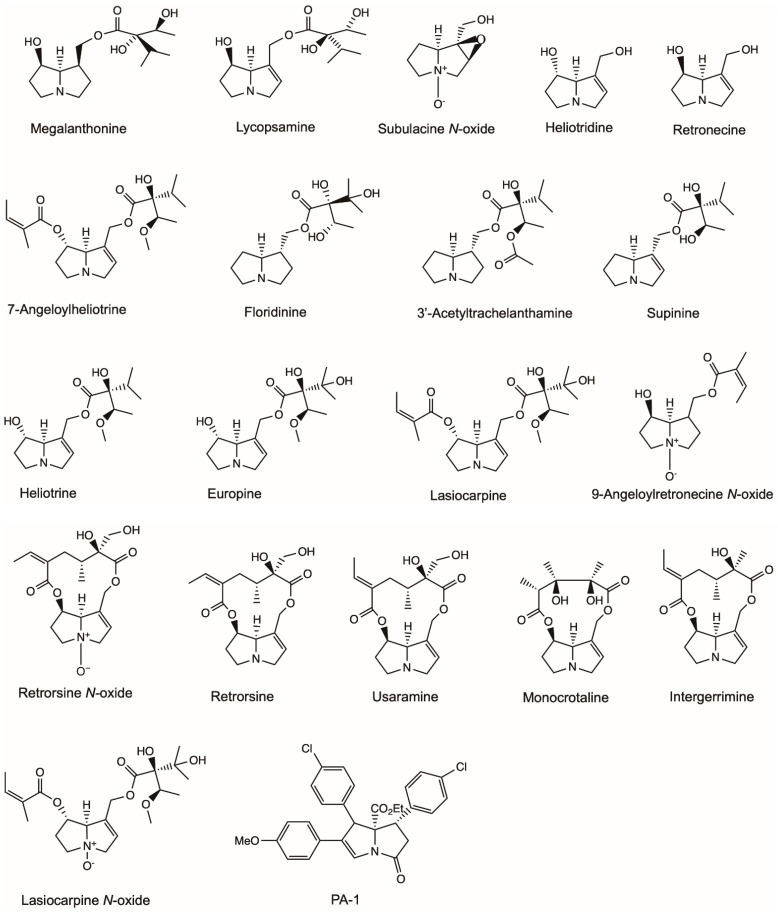
Structures of pyrrolizidine alkaloids that showed anti-microbial activity in this review.

Europine, heliotridine, lasiocarpine and lasiocarpine *N*-oxide were identified from the aerial parts of *Heliotropium ellipticum*, and their anti-microbial activities were investigated (Figure 3) [28]. Europine, lasiocarpine and lasiocarpine *N*-oxide showed anti-bacterial activity to *Escherichia coli* and *Enterobacter cloacae*. Europine had anti-fungal activities to *Aspergillus flavus*, *Drechslera tetramera* and *Fusarium moniliforme*. Heliotridine had anti-fungal activities to *D. tetramera*, and lasiocarpine *N*-oxide had anti-fungal activities to *Curvularia lunata* and *F. moniliforme*.

9-Angeloylretronecine, supinine and lasiocarpine isolated from the aerial parts of *Heliotropium bursiferum* had antimicrobial activity to *Bacillus subtilis* and phytopathogenic fungi *Candida tropicalis* and *Aspergillus niger* at a dose of 50 mg/mL (Figure 3) [29].

Singh et al. [30] isolated subulacine *N*-oxide, 7-Angeloylheliotrine, retronecine and heliotrine from *H. subulatum* (Figure 3). All four isolated PAs demonstrated pronounced activities to the selected microorganisms, including bacteria (*E. coli*, *Streptococcus pneumoniae*, *B. subtilis*, *Bacillus anthracis* and *Staphylococcus aureus*) and fungi (*Aspergillus fumigatus*, *A. niger*, *Rhizoctonia phaseoli* and *Penicillium chrysogenum*). 7-Angeloylheliotrine possessed maximum activity to *E. coli* and *P. chrysogenum* with a total inhibition area of 16.13 ± 0.116 mm and 11.61 ± 0.268 mm, respectively.

Da Silva Negreiros Neto, et al. [31] investigated the anti-biofilm and antibacterial activities of PAs isolated from *Crotalaria* genus (Figure 3). Usaramine isolated from the seeds of *Crotalaria pallida* demonstrated an anti-biofilm activity against *Staphylococcus epidermidis* by reducing more than 50% of biofilm formation without killing the bacteria. Therefore, usaramine could be assumed as a prototype for the development of new anti-biofilm molecules for pharmaceutical and industrial purposes. Monocrotaline obtained from *Crotalaria retusa* seeds showed a significant inhibition of the parasite *Trichoderma vaginalis*, killing 74% of parasite cells at a concentration of 1 mg/mL. However, retronecine showed no anti-activity to *T. vaginalis* at all concentrations, whereas its semi-synthetic derivative azido-retronecine was more active than monocrotaline by killing 85% of *T. vaginalis* at 1 mg/mL.

Li et al. [32] investigated the anti-microbial activity (Figure 3) of a novel PA (PA-1), which was synthesized by organocatalytic domino reaction [33]. PA-1 exhibited predominantly strong antibacterial activity towards all six bacteria (*S. epidermidis*, *S. aureus*, *B. subtilis*, *E. coli*, *Proteus vulgaris*, *Pseudomonas aeruginosa*) and two fungi (*A. niger*, *Candida albicans*) tested with minimum bactericidal concentration (MIC) values ranging from 0.0039 to 0.025 mg/mL. PA-1 killed all *E. coli* and *S. aureus* completely at 2 MIC (0.0078–0.05 mg/mL) within eight hours. PA-1 exerted its antibacterial activity by acting on membrane phospholipids and phosphate groups and then damaging the structures of cellular membrane, finally leading to cell death.

Megalanthonine and lycopsamine were isolated from *Heliotropium megalanthum* and their anti-fungal effects were evaluated (Figure 3) [34]. Both PAs inhibited the mycelial growth of plant pathogen *F. moniliforme* at a dose of 0.5 mg/mL. Europine isolated from *Heliotropium bovei* also showed significant anti-fungal activity to *F. moniliforme* in a dose series ranging from 0.01 to 0.25 mg/mL [35]. 3′-Acetyltrachelanthamine and floridinine isolated from *Heliotropium floridum* had anti-fungal activities to *F. oxysporum*, *F. moniliforme*, *F. avenaceum* and *F. solani* at a dose of 0.5 mg/mL [36].

Hol and Van Veen [37] investigated the growth-reducing effects of PAs from *Jacobaea vulgaris* on nine plant-associated fungi. The growth of five soil fungal strains of *F. oxysporum*, *F. sambucinum* and *Trichoderma* sp. was temporarily inhibited by different purified PAs, including monocrotaline, retrorsine, retrorsine *N*-oxide and a PA plant extract (Figure 3). The strongest inhibitory effects were found at the concentrations 0.33 and 3.33 mM, which were comparable to PA concentrations (0.3–3 mM fresh weight) found in plant tissue under natural conditions for *J. vulgaris* [38,39]. In another study by Hol [40], integerrimine, monocrotaline, retrorsine and retrorsine *N*-oxide (Figure 3) isolated from *Senecio brasiliensis* inhibited the growth of fungi (*F. sambucinum*, *Mortierella* sp., *Minimedusa* sp., *Plectosphearella cucumerina*, *Rhizoctonia* sp., *Broomella acuta*, *Pestalotiopsis* sp. and *Trichoderma* sp.) at a concentration between 0.01 and 1.08 mg/mL.

### 2.2. Anti-Inflammatory Activity

The inflammatory process is a physiological response of the body in order to eliminate, neutralize and destroy stimuli resulting from infection or tissue damage [41]. Diseases usually involve an ongoing inflammatory response, such as that observed in atherosclerosis and cancer [42,43]. Inflammation is a critical component of tumor progression, and many cancers arise from sites of infection, chronic irritation and inflammation [42].

**Figure 4:**
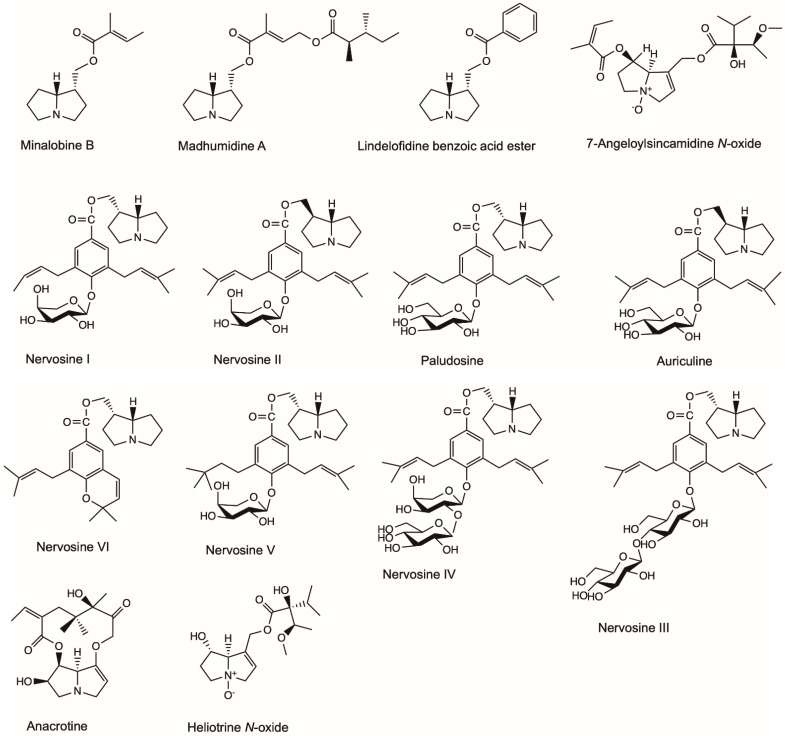
Effective chemical structures of pyrrolizidine alkaloids with anti-inflammatory activities in this review.

The popular murine macrophage cell line, RAW 264.7, is often used to screen natural products for bioactivity and to predict their potential effect in vivo or on primary cells [44]. The murine macrophage cell line’s response is considered to reflect the potential *de novo* response of humans and is used to evaluate the effective bioactivity of tested compounds [45]. Madhumidine A, lindelofidine benzoic acid ester and minalobine B isolated from the leaves of *Madhuca pasquieri* had anti-inflammatory activity against lipopolysaccharide-induced nitric oxide (NO) production in macrophage RAW 264.7 (Figure 4) [46]. Heliotrine, heliotrine *N*-oxide, 7-Angeloylsincamidine *N*-oxide and europine isolated from the aerial parts of *Heliotropium digynum* were evaluated against the NO production in lipopolysaccharide-induced murine macrophages RAW 264.7 cells [47]. The IC_50_ values (the concentration of compound required for 50% inhibition) of the four compounds were 52.4, 85.1, 105.1 and 7.9 µM, respectively [47]. Nervosine I, nervosine II, paludosine, auriculine, nervosine III, nervosine IV, nervosine V and nervosine VI isolated from *Liparis nervosa* showed strong inhibitory activities against lipopolysaccharide-induced NO production in RAW 264.7 macrophages with IC_50_ values in the range of 2.16–38.25 µM, thus showing potential anti-inflammatory activity [48].

Oedema is an excess of fluid in the tissues and is a symptom of disease [49]. Ghosh and Singh [50] investigated the anti-inflammatory activity of anacrotine isolated from *Crotalaria laburnifolia* Linn in rats (Figure 4). Anacrotine (40 mg/kg s.c. * 5 alternate days) had no significant inhibitory effect against formalin-induced arthritis, while it showed inhibition against carrageenin-induced oedema. In normal or adrenalectomized rats, anacrotine (10 mg/kg s.c.) inhibited hyaluronidase-induced oedema. Anacrotine (40 mg/kg s.c. and 30 mg/kg i.p., respectively) was ineffective against 5-hydroxytryptamine- and dextran-induced oedema; however, it was effective against bradykinin- and prostaglandin-induced oedema (in a dose of 20 mg/kg i.p.). Anacrotine also showed inhibitory effects against cotton-pellet granuloma.

### 2.3. Antiviral Activity

Viruses are major pathogenic agents causing a variety of serious disease in humans, other animals and plants [51]. Antiviral drugs are most commonly designed with the purpose of combating various viral infections, such as human immunodeficiency virus (HIV) and influenza [52,53].

The ethanol and hexane crude extract of *H. subulatum* showed significant antiviral activity to *Coxsackie*, *Poliomyelitis* and *Measles* at 500 and 100 µg/mL concentrations. Heliotrine and 7-Angeloylheliotrine isolated from this plant species possessed activity against *Poliomyelitis* and *Vesicular stomatitis* virus at a concentration of 10 µg/mL [54].

**Figure 5:**
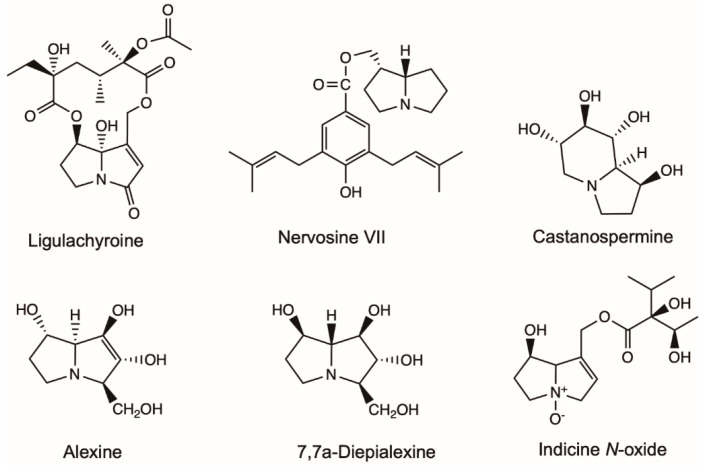
Effective chemical structures of pyrrolizidine alkaloids with anti-cancer or anti-virus activities in this review. Structures of lycopsamine, retronecine, heliotrine and 7-Angeloylheliotrine with anti-cancer activities were listed in Figure 3.

Alexine isolated from *Alexa leiopetala* and four stereoisomers isolated from *Castanospermum austral* were investigated for inhibitory activity against the growth of HIV-1 (Figure 5). Five alexines were separated, among which only 7,7a-Diepialexine restricted virus growth with IC_50_ 0.38 mM. The antivirus effects of 7,7a-Diepialenxine correlated with the inhibitory activity against purified pig kidney α-glucosidase 1 of the glycoprotein processing enzymes and the reduced cleavage of the precursor HIV-1 glycoprotein gp160 [55].

### 2.4. Antineoplastic Activity

Cancer is the second leading cause of death worldwide, with 9.6 million cancer-related deaths in 2018 [56]. The global health burden attributable to cancer has increased substantially over the past three decades, leading to a rise in the prescription of chemotherapeutic drugs [57,58]. The development of antineoplastic compounds is, therefore, urgent.

Leukemia is a broad term for cancers of the blood cells [59]. Indicine *N*-oxide was used in the treatment of leukemia (Figure 5). Twenty-two patients with refractory acute leukemia received indicine *N*-oxide daily for five consecutive days in a dose-seeking study [60]. Of eight patients with refractory acute lymphocytic leukemia, one had a complete remission, and one had a partial remission. Of 11 patients with refractory acute nonlymphocytic leukemia, two patients had complete remissions. Of three patients with blast crisis of chronic granulocytic leukemia, one patient had a partial remission. Five patients had severe hepatic toxicity, probably due to veno-occlusive disease induced by indicine *N*-oxide. Miser et al. [61] treated 31 children with acute lymphoblastic leukemia (ALL), 14 children with acute non-lymphoblastic leukemia (ANLL) in relapse, and one child with chronic myelogenous leukemia (CML) in blast crisis (CALLA negative) with indicine *N*-oxide in a Phase II study. The efficacy and toxicity of the drug were assessed at two dose levels: 2000 mg/m^2^/day for 5 consecutive days (14 patients) and 2500 mg/m^2^/day for five consecutive days (17 patients). One patient with ALL at each dose level achieved a complete response (CR) lasting six months and one month, respectively. The patient with CML achieved a partial response lasting four months. None of the patients with ANLL achieved a CR. Hepatotoxicity was mild (grade 1 or 2) in 63% and moderate (grade 3) in 9% of patients; three patients (9%) experienced severe hepatotoxicity.

*Ligularia achyrotricha* (Diels) Ling has been used as a traditional Tibetan herbal medicine for a long time for treating dermatosis, diphtheria and pestilence [62]. Hua, et al. [63] isolated ligulachyroine A from the roots of *L. achyrotricha* (Figure 5), a new twelve-membered macrocyclic PA with a ketonic group located at C-3 and a hydroxy group linked at C-8. It exhibited moderate activity against a human leukemia cell (HL-60) and a human hepatoma cell (SMMC-7721) [63].

Nervosine VII isolated from the plant of *L. nervosa* induced autophagy and apoptosis in HCT116 human colorectal cancer cells by the activated MAPKs signaling pathway, including JNK, ERK1/2 and p38, and suppressing the p53 signaling pathway (Figure 5) [64]. Nervosine VII-induced apoptosis associated with the intrinsic pathway by the activation of caspase-9, -3 and -7. The characteristic of autophagy induced by nervosine VII was the regulation of autophagic markers, including the increase in LC3-II and beclin 1 proteins, and the decrease in p62 protein. Those finding indicated that the nervosine VII may be a novel therapeutic method for the treatment of cancer.

Lycopsamine showed significant antiproliferative effects in A549 lung cancer cells in a dose-reliant manner (Figure 5) [65]. The antiproliferative effects of lycopsamine were associated with its autophagy inducing, apoptosis inducing, and inhibiting IL-2 expression. Overall lycopsamine is a potential anti-lung cancer agent and can be a lead molecule in lung cancer treatment.

Appadurai and Rathinasamy [66] isolated indicine *N*-oxide from *Heliotropium indicum* and found that it inhibited the proliferation of various cancer cell lines in a concentration-dependent manner with IC_50_ ranging from 46 to 100 μM. It blocked the cell cycle progression at mitosis without significantly altering the organization of the spindle and interphase microtubules. The toxicities of indicine *N*-oxide at higher concentration was due to DNA-damaging effects and depolymerization of microtubules. Indicine *N*-oxide bound to tubulin at a distinct site, so it could serve as a template for the synthesis of some potent analogs for the treatment of cancer.

Heliotrine, 7-Angeloylheliotrine, retronecine, subulacine and subulacine *N*-oxide were isolated from *H. subulatum* (Figure 5) [54]. The 7-Angeloylheliotrine and retronecine showed activity at 5 µg/kg/day of 41.7% and 38.6% inhibition against Sarcoma 180. The hexane extract (3 µg/mL) and 7-Angeloylheliotrine (10 and 5 µg/mL) showed selective cytotoxicity against Chinese hamster V_79_ cells.

### 2.5. Acetylcholinesterase Inhibitory Activity

Acetylcholinesterase (AChE) is a serine hydrolase that terminates the action of the neurotransmitter acetylcholine by hydrolyzing it into acetic acid and choline [67]. The AChE inhibitors bind to the enzyme and interfere with the breakdown of acetylcholine, leading to the deposition of acetylcholine in the nerve synapses and causing disrupted neurotransmission [68]. Cholinesterase inhibitors increase parasympathetic nervous system (cholinergic) activity indirectly by inhibiting AChE, thereby preventing the breakdown of acetylcholine. The AChE inhibitors are used in the treatment of Alzheimer’s disease [69], the treatment and diagnosis of myasthenia gravis [70] and the treatment of diabetic urinary bladder [71].

**Figure 6:**
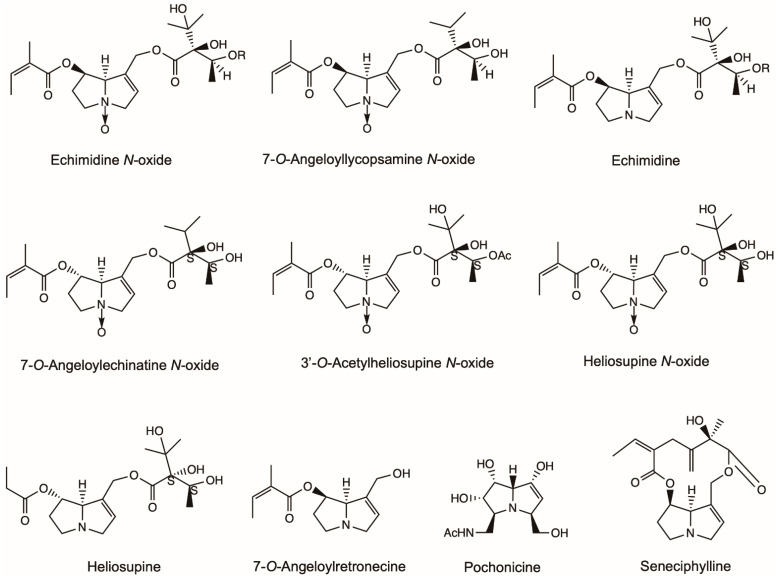
Structures of pyrrolizidine alkaloids (PAs) with acetylcholinesterase inhibitory activity and miscellaneous activity discussed in this review. Except seneciphylline and pochonicine, the other PAs represent the acetylcholinesterase inhibitors in this figure. Heliotrine, integerrimine, retrorsine, senecionine and usaramine also had anti-ulcer effects and were listed in Figure 1.

7-*O*-Angeloyllycopsamine *N*-oxide, echimidine *N*-oxide, echimidine and 7-*O*-Angeloylretronecine extracted from the whole plant of *Echium confusum* Coincy showed moderate activities in inhibiting AChE with IC_50_ (sample concentration required to inhibit the hydrolysis of the substrate by 50%) 0.276–0.769 mM (Figure 6) [72]. 7-*O*-Angeloylechinatine *N*-oxide, 3′-*O*-Acetylheliosupine *N*-oxide, heliosupine *N*-oxide and heliosupine extracted from the whole plant of *Solenanthus lanatus* showed inhibition activity against the AChE with IC_50_ 0.53–0.60 mM (Figure 6) [73]. 

### 2.6. Miscellaneous Activity

Besides the biological activities of PAs summarized above, other pharmacological properties of PAs were also detected, such as anti-ulcer and ganglion blocking.

Heliotrine isolated from the seeds of *H. indicum* was found to possess ganglion blocking activity [74]. Integerrimine, retrorsine, senecionine, usaramine and seneciphylline obtained from *S. brasiliensis* showed significant antiulcerogenic activity in both acute and chronic gastric ulcers on rats (Figure 1 and Figure 6) [75]. The PA extract increased both the levels of gastrin and the expression of Epidermal Growth Factor (EGF) after chronic treatment. Furthermore, the exfoliation of superficial cells, hemorrhages and blood cell infiltration reduced in histological examinations. A mixture of PAs consisting of senecionine, integerrimine, retrorsine, usaramine and seneciphylline were extracted from *S. brasiliensis* inflorescences and presented a significant anti-ulcer effect (Figure 1 and Figure 6) [76]. The lesion induced by indomethacin-bethanechol and hypothermic-restraint-induced gastric ulcer was significantly inhibited by PA. PA extracts also showed significant inhibition in the cysteamine-induced duodenal ulcers. Pochonicine, a polyhydroxylated PA, was isolated from a solid fermentation culture of the fungal strain *Pochonia suchlasporia* var. suchlasporia TAMA 87. It demonstrated potent inhabitation against β-*N*-Acetylglucosaminidases (GlcNAcases) of various organisms including insects, fungi, mammals and a plant (Figure 6) [77].

## 3. Recommendations for Medication

### 3.1. Restrictions on the Daily and Cumulative Dosage

The Germany Federal Health Bureau defined, in 1992, the maximum daily intake of PAs for internal use as limited to 1 μg for a maximum of 6 weeks/year and 0.1 μg for medicines with no limited duration of treatment [78]. In Belgium, it was proposed that the limit for PAs in herbs was set at 1 ppm in 2001 [79]. The provisional tolerance daily intake for PAs in humans was set up by the Australia New Zealand Food Authority (ANZFA) at 1 μg/kg body weight/day in 2001 [80]. With regard to the mutagenic effects of PAs, the Dutch National Institute for Public Health and the Environment (RIVM) stated that a virtually safe dose for PAs would be 0.00043 μg/kg body weight/day in 2005 [81]. The British “Committee on Toxicity of Chemicals in Food, Consumer Products and Environment” (COT) evaluated the non-cancer effects of PAs and concluded that doses of PAs below 0.007 μg/kg body weight/day would unlikely be concerning in 2014 [82]. Accordingly, the German Federal Institute for Risk Assessment (BfR) also defined a safe dose of 1, 2-unsaturated PAs of 0.007 μg/kg (0.42 μg/60 kg adult) for daily intake [81]. The Committee on Herbal Medicinal Products (HMPC) of the European Medicines Agency (EMA) stated that the daily oral administration from herbal medicinal products and food was limited to 0.35 μg for a maximum of 14 days [83]. 

Many countries and authorities have set various limitations for PAs as mentioned above; however, there is no international standard. All medicines have toxicity to some degree. It is crucial to clearly indicate the dosage of medicines containing PAs.

### 3.2. Risks of Medication

The Consumer Healthcare Products Association (CHPA) recommend that all products with botanical ingredients containing PA should bear the following cautionary statement on the label: “For external use only. Do not apply to broken or abraded skin. Do not use when nursing” [84]. In Germany, the Federal Ministry of Health required that all the herbal medicines containing PAs must meet the regulations, and the package needs to contain the warning notice, “Do not use during pregnancy and lactation” [83,85]. There are similar regulations in the use of herbal medicines in Austria and Switzerland [83,86]. In January 2004, selling the oral drug containing *Senecio* plants was banned in the United Kingdom [87]. In 2001, the US Food and Drug Administration (FDA) banned the use of comfrey in dietary supplements [88].

Additionally, manufacturers should warn the patients not to drink alcohol during the period using PA-containing medicines [89]. When patients take PA-containing medicines, drugs that have hepatoxic synergistic effects are not allowed to be taken at the same time [90].

## 4. Conclusions and Perspectives

PAs are a widespread group of secondary metabolites. Although PAs have potential health risks to humans, they also demonstrate many beneficial pharmacy properties, such as anti-bacterial, anti-fungi, anti-inflammatory, anti-cancer and anti-virus properties. To date, the main pharmaceutical application of PAs is in traditional medicine with the PA-containing plant extracts as ingredient. There are no international safety standards for screening and quality control of PA-containing plants in herbal medicine and food supplements. Most results of the pharmaceutical activities of PAs were derived from laboratory studies and in vitro tests; thus there remains a lack of clinical evidence.

Though several countries and organizations established regulations to restrict the exposure to PA-containing food and herb medicines to avoid the toxicity, these regulations are based on case studies and cannot be universally applied to all PAs. It is difficult to determine a toxic dosage threshold for different PAs that may have varying toxicological effects. Therefore, a systematic assessment system with an international standard is urgently needed for predicting the toxicity of PA-containing foods and medicinal herbs.

With the development of analytic methods and chemistry strategies [91], it is of great importance to maintain the bioactivity and reduce the toxicity of PAs. The pharmacological results of PAs need to be confirmed in a consistent methodology and with more plant species under a universal assessment system. Regardless of this, it cannot be denied that PAs have beneficial biological properties, and the prospect of pharmacy application is promising.
